# Screening of Phenolic Antioxidant Components in *Polygonum perfoliatum* L. Using LC×LC-ECD and UPLC-Orbitrap-MS/MS

**DOI:** 10.3390/molecules31203612

**Published:** 2026-10-10

**Authors:** Wei Pan, Xing-Rui Li, Hai-Yan Gong, Zheng-Yan Tan, Pei Xu, Piao Wang, Xing-Qin Luo, Cheng Yang, Ya-Ping Zhou, Rong-Xiang Chen

**Affiliations:** 1 School of Pharmacy, Zunyi Medical University, Zunyi 563000, China; 2 School of Basic Medical Sciences, Zunyi Medical University, Zunyi 563000, China

**Keywords:** *Polygonum perfoliatum* L., offline comprehensive two-dimensional liquid chromatography, electrochemical detection, phenolic acids, flavonoids, quality evaluation

## Abstract

*Polygonum perfoliatum* L. is a medicinal plant rich in phenolic and flavonoid compounds, but severe peak overlap in one-dimensional liquid chromatography (1D-LC) hinders the separation of antioxidants. We developed an offline comprehensive two-dimensional liquid chromatography-electrochemical detection (LC×LC-ECD) platform with high orthogonality and sensitive electrochemical response for screening of antioxidants. Identification was performed using ultra-high-performance liquid chromatography-high-resolution tandem mass spectrometry (UHPLC-Orbitrap-MS/MS), and quantification was performed using high-performance liquid chromatography with electrochemical detection (HPLC-ECD). A total of 63 antioxidants were annotated, mainly phenolic acids and flavonoids, with caffeoyl and quercetin derivatives most abundant. The first-dimension fractions exhibited radical scavenging capacity, and their peak areas were positively correlated with the activity, with correlation coefficients of 0.9697 (*p* < 0.001) for 2,2′-azino-bis (3-ethylbenzothiazoline-6-sulfonic acid) diammonium salt (ABTS) and 0.9688 (*p* < 0.001) for 1,1-diphenyl-2-picrylhydrazyl (DPPH). The 11 major compounds were quantitatively analyzed. Tartroylcaffeic acid and quercetin 3-*O*-*β*-*D*-glucuronide predominated, with average contents of 5101.7–29,296.2 µg/g and 3876.9–21,535.2 µg/g, respectively. Multivariate statistical analysis indicated that geographical origin influenced compound profiles, highlighting the need for source-specific evaluation. This offline LC×LC-ECD strategy overcomes the separation bottlenecks of 1D-LC in complex plant extracts and provides a new paradigm for systematic antioxidant screening and activity-guided separation.

## 1. Introduction

*P. perfoliatum* is a perennial herbaceous vine of the Polygonaceae family [1]. Its aerial parts, including stems, leaves and fruits, are all medicinal materials, which have been officially recorded in the 2025 edition of the *Chinese Pharmacopoeia* [2]. With a long history of medicinal application in China, *P. perfoliatum* is abundant in natural resources and widely distributed in Jiangsu, Guangdong, Sichuan, Guizhou and other regions. It is also found in other Asian countries, such as Bhutan, India, Japan and South Korea [3].

In addition to its medicinal uses, the plant can be processed into functional food and serves as a high-quality feed for livestock and poultry [4]. Modern pharmacological studies indicate that *P. perfoliatum* exhibits diverse pharmacological activities, including antiviral, antioxidant, and antibacterial properties [5]. It demonstrates significant efficacy in cough suppression, tumor inhibition, and antibacterial effects [6]. In clinical practice, it is often used to treat various conditions such as analgesia, inflammation reduction, and hepatoprotection [7]. The chemical composition of *P. perfoliatum* is complex, rich in phenolic acids, flavonoids, coumarins, lignans, polyphenols, phenylpropanoids, etc. [8]. Flavonoids are the primary chemical components of *P. perfoliatum*. Currently reported major components include kaempferol, quercetin, quercetin-3-*O*-*β*-*D*-glucuronide, kaempferol-3-*O*-rutinoside, and rutin [9,10].

HPLC coupled with ultraviolet detection is commonly used for the quantitative analysis of phenolic acids and flavonoids [11]. However, its selectivity and sensitivity are insufficient for the analysis of trace phenolics [12]. In recent years, HPLC coupled with electrochemical detection (ECD) has been widely used to analyze electroactive compounds in various samples due to its excellent selectivity and sensitivity [13]. Compared to traditional UV and DAD detection, ECD can recognize changes in the electrical signals of substances and selectively detect compounds with oxidation or reduction capabilities [14]. In particular, phenolic acids and flavonoids often exhibit superior response values compared to compounds lacking phenolic hydroxyl groups [15]. HPLC-ECD has been extensively applied in the screening of active ingredients and quantitative determination of phenolic compounds in food and pharmaceuticals, and its response value can directly reflect the antioxidant capacity of the compounds [16,17]. Since phenolic acids and flavonoids are key components of *P. perfoliatum*, the reliable determination of phenolic compounds using HPLC-ECD is a highly promising method for assessing the quality and grading of *P. perfoliatum*.

Limited by low peak capacity, one-dimensional liquid chromatography (1D-LC) often causes chromatographic peak crowding and unavoidable peak overlap in the separation of complex samples [18]. In contrast, two-dimensional liquid chromatography (2D-LC) achieves a dramatic improvement in peak capacity, enabling simultaneous separation and analysis of complex multicomponent mixtures [19]. 2D-LC can be divided into online and offline modes. The online mode features short analysis times and a high degree of automation [20], whereas in the offline mode, the eluate from the first dimension must be collected within a short timeframe before undergoing independent analysis in the second dimension [21].

Compared to online multi-heart-cutting two-dimensional liquid chromatography, comprehensive two-dimensional liquid chromatography (LC×LC) can more comprehensively characterize the complex compound composition in plant samples. During the separation process in the second dimension, performance is not limited by analysis duration, and conditions can be optimized to improve separation efficiency, such as using columns with higher separation efficiency [22,23]. Additionally, fractions can be used for separate mass spectrometry identification and antioxidant activity assays [24]. Collectively, LC×LC significantly enhances peak capacity and resolution [25], enabling the efficient resolution of complex compounds with a wide range of polarities and similar structures [26]. The combination of LC×LC further expands the application scope of HPLC-ECD in natural product analysis. For plant extracts, LC×LC can separate isomers and polymers that are difficult to distinguish by conventional 1D-LC. Combined with directed electrochemical detection, it can precisely identify components with antioxidant activity [21,24]. This hyphenated strategy not only greatly enhances the separation efficiency of complex samples, but also realizes rapid component identification and functional verification through multi-dimensional data correlation [27], including retention time, electrochemical response and mass spectrometry information, providing strong technical support for the deep development of natural products [28,29].

Against this background, the present study aims to establish an offline LC×LC-ECD (Thermo Fisher Scientific, Waltham, MA, USA) and UHPLC-Orbitrap high-resolution mass spectrometry (Thermo Fisher Scientific, Waltham, MA, USA) (LC×LC-ECD/UHPLC-Orbitrap-MS/MS) analytical platform for the activity-guided antioxidant analysis of *P. perfoliatum*. Specifically, this study intends to resolve the peak overlap and low identification accuracy issues of traditional one-dimensional chromatographic methods for complex phenolic components in *P. perfoliatum*, quantitatively analyze the key bioactive phenolic constituents, and clarify the radical-scavenging contribution of different phenolic fractions. The findings of this study are expected to establish a reliable technical approach for the efficient separation and functional identification of bioactive components in *P. perfoliatum*, and provide a theoretical and technical basis for its quality evaluation and in-depth development and utilization.

## 2. Results and Discussion

### 2.1. LC×LC-ECD Separation and UPLC-Orbitrap-MS/MS Identification

**Figure 1:**
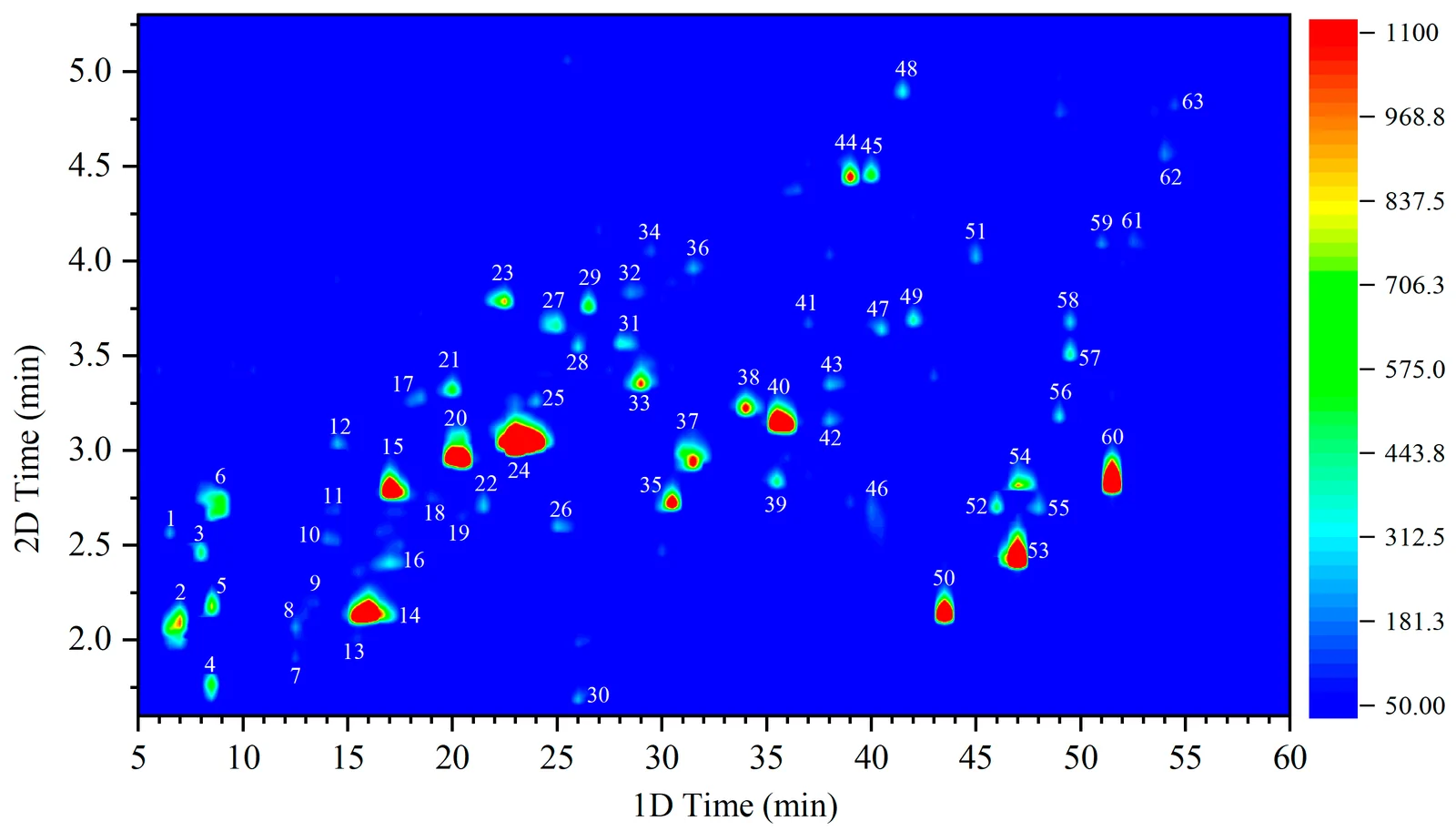
Offline LC×LC-ECD chromatogram of *P. perfoliatum* extracts. Peaks 1–63: 63 major peaks of *P. perfoliatum*.

Compared to 1D-LC, LC×LC offers significant advantages in peak capacity and resolution, making it particularly suitable for the separation of complex samples [30]. In this study, stationary phases and mobile phases with different selectivities were used in the first and second dimensions, respectively. The stationary phases were phenylhexyl and C18, and the mobile phases were methanol and acetonitrile, thereby achieving a substantial increase in peak capacity. LC×LC was used to separate the chemical components of *P. perfoliatum*. Combined with the selective detection capability of ECD for antioxidant components, the first-dimension fractions were further separated and characterized in the second dimension. The LC×LC chromatogram obtained under the specified conditions is shown in Figure 1. The orthogonality and peak capacity of the constructed LC×LC system were evaluated using the calculation methods proposed by Camenzuli et al. [31,32,33]. Sixty-three identified components were selected, and their retention times in both dimensions were recorded. The orthogonality was calculated to be 63.72%, indicating good orthogonality of the two-dimensional separation. The peak capacities of the first- and second-dimension LC were 67 (average peak width 0.82 min) and 23 (average peak width 0.30 min), respectively. Therefore, the theoretical peak capacity of the two-dimensional LC system was 1568, and the effective peak capacity was 1051. These results fully demonstrate the advantages of LC×LC in the separation of complex samples.

Fractions from ^1^D-LC were analyzed by UPLC-Orbitrap-MS/MS, achieving component separation and identification. Based on the relative molecular mass (*m*/*z*) and fragment ions, a total of 63 compounds were annotated. Among these 63 compounds, 10 were unambiguously identified by comparison with authentic standards, together with precursor ion and fragment information and assigned as Level I identification. The remaining 53 compounds were tentatively identified via precursor ion, fragment information and database matching and assigned as Level II identification, as shown in Table 1. The overlaid TIC chromatograms of these 63 compounds are provided in the Supplementary Materials (Figure S1).

These 63 compounds comprised 19 glycosides, 20 phenolic acids, 6 proanthocyanidins, 17 flavonoids, and 1 amino acid. The glycosides included glucogallin isomers 1–5, dihydroxybenzoic acid glucoside and its isomer, koaburaside, vanillic acid -*O*-*β*-*D*-glucopyranoside, hydroxyphenylacetic acid glucoside, caffeoyl-*β*-*D*-glucose, *p*-coumaric acid glucoside, di-*O*-galloyl-*β*-*D*-glucopyranose, sinapic acid glucoside and its isomer, ferulic acid-*O*-*β*-*D*-glucoside, helonioside B, smiglaside C, and dihydro-tri-*O*-*p*-coumaroylsucrose. The phenolic acids included gallic acid, caffeoyltartaric acid isomers, *p*-coumaroyltartaric acid isomers, fertaric acid isomers, dihydroxybenzoyl-trihydroxyphenylacetic acid (two isomers), caffeoylglutaric acid, chicoric acid, chlorogenic acid, and 1,5-dicaffeoylquinic acid. The proanthocyanidins included procyanidin B isomers 1 and 2, procyanidin B2, procyanidin C and its isomer, and 2×(E)C-GA. The flavonoids included isoglucodistylin, catechin, epicatechin, hydroxyflavanone glucoside, teatannin, Galloylcatechin, kaempferol-3-*O*-arabinoside, taxifolin, epicatechin 3-*O*-(4-*O*-methylgallate), myricitrin, quercetin-3-*O*-*β*-*D*-Glucuronide, hyperin, quercetin-7-*O*-*β*-*D*-Glucuronide, avicularin, luteolin-7-*O*-glucuronide, trifolin, and quercetin. The single amino acid was Trp. The main chemical constituents of *P. perfoliatum* are flavonoids and phenolic acids: the more polar fraction consists primarily of proanthocyanidins and monocaffeoyltartaric acid, accompanied by *p*-coumaroyltartaric acid, chlorogenic acid, etc., while weakly polar fractions were dominated by quercetin and its glycoside derivatives.

Three isomers with molecular formula C_13_H_12_O_9_ were detected in *P perfoliatum*. by LC-HRMS, all yielding [M − H]^−^ at *m*/*z* 311.041 (mass error < 3 ppm). Peak 20 was unambiguously identified as *trans*-caffeoyltartaric acid using a commercial standard, with its MS/MS spectrum dominated by tartrate anion *m*/*z* 149.009 (base peak) and low-abundance caffeate fragment *m*/*z* 179.035. Peak 14 eluted earlier and was tentatively assigned as *cis*-caffeoyltartaric acid; it shared the base peak *m*/*z* 149.009 with the trans form but showed elevated relative intensities of caffeate-derived ions (*m*/*z* 179, 135), consistent with the higher polarity of the cis isomer in reversed-phase separation. The latest-eluting Peak 24 was tentatively characterized as tartroylcaffeic acid, a regioisomer with reversed ester linkage. Its MS/MS spectrum featured caffeate anion *m*/*z* 179.035 as the base peak, indicating the caffeic acid moiety bore the free carboxyl group, and its reduced polarity accounted for the longer retention time. Detailed information is provided in Figure S3 (Supplementary Materials). Owing to the largest peak area of Peak 24, the corresponding monomer was isolated (purity > 98%, HPLC) for subsequent quantification.

ECD is one of the effective methods for evaluating antioxidant activity. Based on charge transfer theory, ECD signals directly reflect the reducing capacity of analytes. When combined with chromatographic separation, it allows for the direct identification of active components [34]; in turn, UHPLC-Orbitrap-MS/MS provides accurate mass and MS/MS fragmentation information for compound identification. By combining these two techniques, the electrochemical response can be correlated with the separated fractions for preliminary identification. Therefore, the hyphenation of LC×LC-ECD with UHPLC-Orbitrap-MS/MS provides a powerful strategy for activity-guided antioxidant screening in *P. perfoliatum*.

### 2.2. Antioxidant Activity of Different Fractions

**Figure 2:**
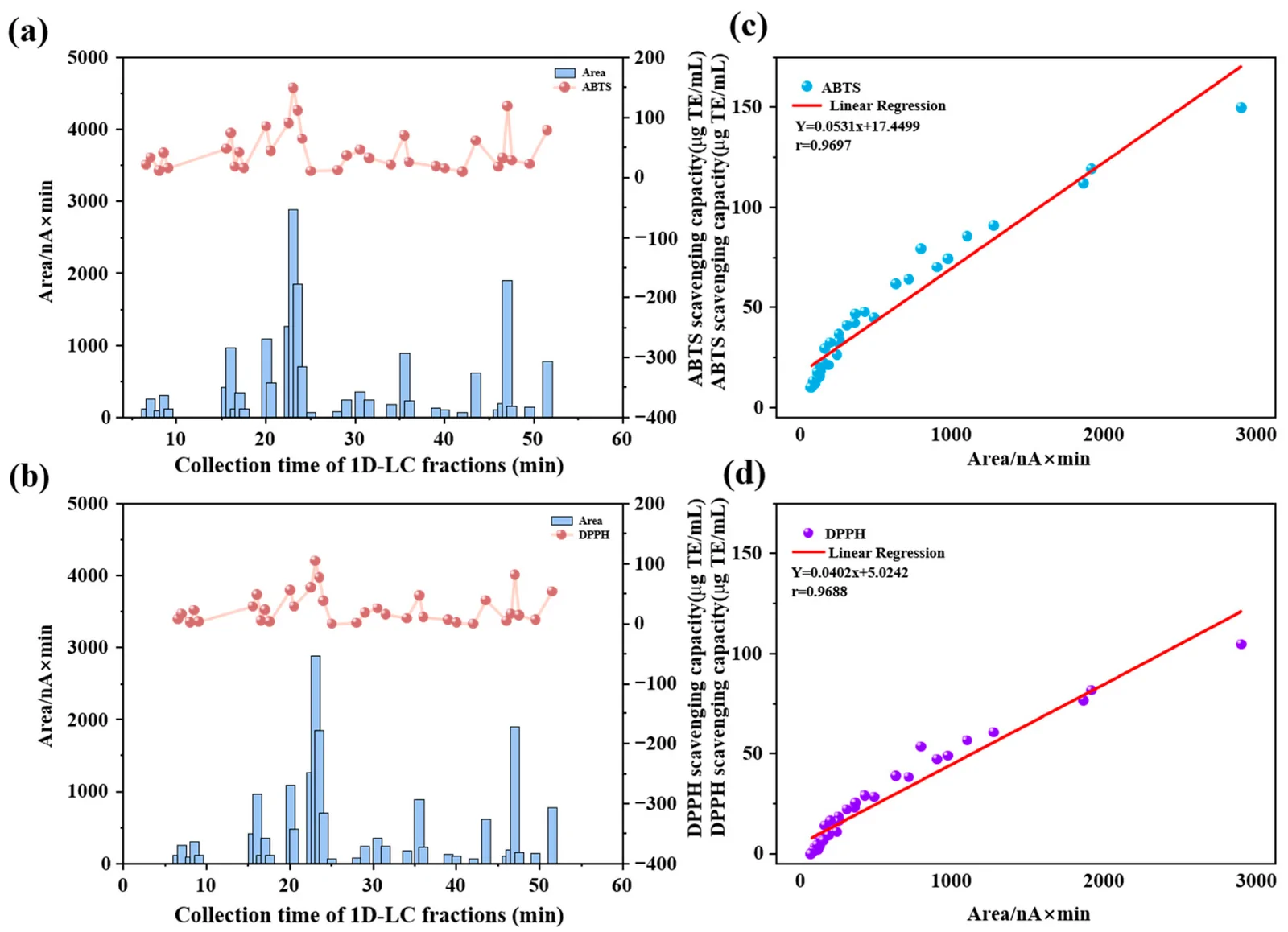
ABTS (**a**,**c**) and DPPH (**b**,**d**) radical scavenging activities of ^1^D-LC fractions from *P. perfoliatum* and their correlation with ECD peak areas.

To verify the antioxidant activity of the screened compounds, the ABTS and DPPH radical scavenging capacities of different fractions obtained by ^1^D-LC were evaluated. The results are shown in Figure 2a,b. The results showed a significant positive correlation between the peak areas of the 2D-LC-ECD fractions and their corresponding radical scavenging equivalents. Figure 2c,d show that the correlation coefficients were 0.9697 (*p* < 0.001) for ABTS and 0.9688 (*p* < 0.001) for the DPPH assay, respectively. The positive response of ECD toward the LC fractions ensured a strong correlation between the electrochemical signal and in vitro radical scavenging capacity, validating offline 2D-LC-ECD combined with radical scavenging assays as a potential screening method for antioxidants in *P. perfoliatum*.

Previous studies have confirmed that *P. perfoliatum* possesses excellent antioxidant capacity, but the specific active components remain unclear [2]. In this study, the extract of *P. perfoliatum* was fractionated by 1D-LC, and the redox capacity of each fraction was evaluated, thereby preliminarily elucidating the contribution of different fractions to the in vitro chemical radical scavenging capacity. The results showed that the major active fractions isolated from *P. perfoliatum* exhibited in vitro chemical radical scavenging capacity, among which phenolic acids and flavonoids were the major chemical classes contributing to the in vitro chemical radical scavenging capacity of the tested fractions. Based on the integrated peak areas from ECD and the radical scavenging rates of the corresponding fractions, the fractions containing tartroylcaffeic acid, quercetin-3-*O*-*β*-*D*-Glucuronide, *trans*-caffeoyltartaric acid, and chicoric acid exhibited the strongest in vitro radical scavenging capacity, indicating that they were the major contributors to the in vitro radical scavenging capacity of the tested fractions.

Phenolics are natural antioxidant compounds widely found in plants, containing one or more hydroxyl groups in their structures [35]. Phenolic acids are characterized by single benzene rings with hydroxyl groups, while flavonoids possess a typical C6-C3-C6 skeleton [36]. Polyphenols in plants not only exert antioxidant activity but are also involved in growth regulation, hormonal activity, antibacterial activity, pH regulation, metabolism, and the induction of dormancy [37]. Phenolic acids are widely distributed in *P. perfoliatum*; for example, several mono-caffeoyl tartaric acids account for a significant proportion of the peak area, making them the primary phenolic acid components in *P. perfoliatum*. These compounds not only possess strong antioxidant activity but also exhibit antibacterial and anti-inflammatory effects, making them a potential bioactive raw material with the potential for phenolic acid extraction [38,39].

Another major class of compounds in *P. perfoliatum* is flavonoids, such as quercetin-3-*O*-*β*-*D*-Glucuronide, luteolin-7-glucuronide, quercetin, and epicatechin. Quercetin-3-*O*-*β*-*D*-glucuronide exhibited the strongest antioxidant activity in the in vitro assays. It has been reported that quercetin-3-*O*-*β*-*D*-Glucuronide possesses anti-inflammatory and antiviral functions and can inhibit lipid peroxidation [40]. In vivo experiments have proven its inhibitory effects on xylene-induced ear edema, acetic acid-induced peritoneal permeability and influenza A virus-triggered pulmonary edema [41]. Furthermore, quercetin is an important compound identified in *Polygonum* species and has various medicinal properties including anti-inflammatory, anticancer, antiviral, and therapeutic effects on allergies and cardiovascular diseases [42,43]. The antioxidant capacity of plant polyphenols is positively correlated with the number of -OH groups on their aromatic rings. Simultaneously, their activity can be enhanced through mechanisms such as chelating metal ions, activating antioxidant enzymes, and forming intramolecular hydrogen bonds. In addition to antioxidant activity, polyphenols are involved in various physiological processes, including plant growth regulation and antimicrobial defense [44].

The developed LC×LC-ECD coupling strategy can rapidly screen compounds with redox capacity in *P. perfoliatum*, such as caffeoyltartaric acid isomers, quercetin and its glycosides. Fractions containing these compounds contribute to the in vitro chemical radical scavenging capacity. The obtained results offer reliable methodological evidence and data support for the resource exploitation of *P. perfoliatum* and the formulation of quality standards targeting its bioactive constituents. However, the antioxidant activity evaluated in this study was limited to in vitro chemical assays performed on LC fractions, and further biological studies are needed to demonstrate the pharmacological relevance of the identified potential antioxidants.

### 2.3. Quantitative Determination of 11 Compounds

#### 2.3.1. Method Validation

**Figure 3:**
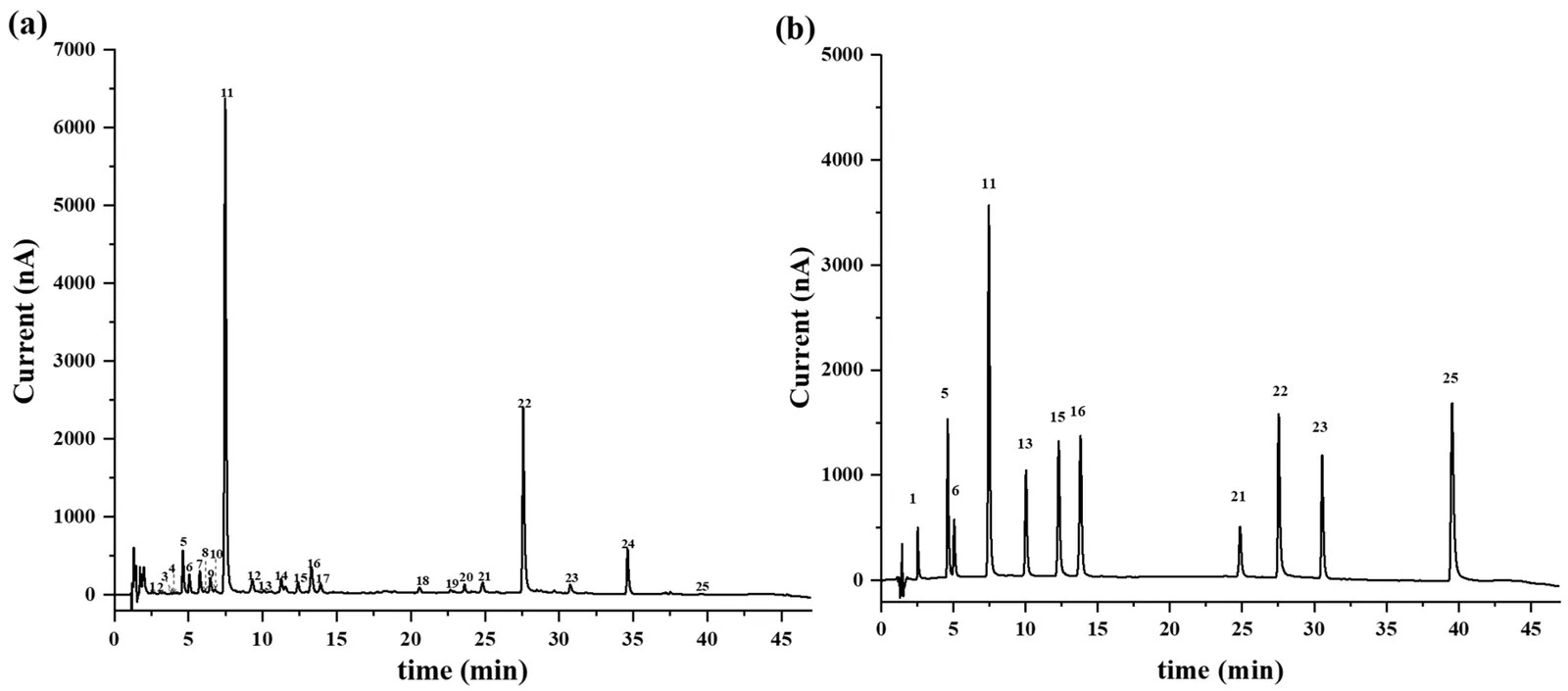
Representative HPLC-ECD chromatograms of *P. perfoliatum* extract (**a**) and a mixed standard solution (**b**). CP1–CP25 are assigned as follows: CP1: gallic acid, CP2: dihydroxybenzoic-acid-glucoside, CP3: procyanidin B isomer 1, CP4: *cis*-caffeoyltartaric acid, CP5: Trp, CP6: *trans*-caffeoyltartaric acid, CP7: catechin, CP8: *p*-coumaroyltartaric acid isomer 1, CP9: procyanidin B isomer 2, CP10: fertaric acid isomer 1, CP11: tartroylcaffeic acid, CP12: procyanidin C isomer 1, CP13: chlorogenic acid, CP14: *p*-coumaroyltartaric acid isomer 2, CP15: epicatechin, CP16:procyanidin B2, CP17: dihydroxyflavanone-glucoside, CP18: galloylcatechin, CP19: kaempferol-3-*O*-arabinoside, CP20: taxifolin, CP21: chicoric acid, CP22: quercetin-3-*O*-*β*-*D*-glucuronide, CP23: 1,5-dicaffeoylquinic acid, CP24: dihydro-tri-*O-p*-coumaroylsucrose, CP25: quercetin.

The linearity, precision, and spiked recovery of the method were evaluated, as shown in Table 2. All compounds showed good linear relationships between concentration and peak area within the detection ranges, with high correlation coefficients (R^2^ > 0.999). For several compounds, such as *trans*-caffeoyltartaric acid, tartroylcaffeic acid and quercetin-3-*O*-*β*-*D*-glucuronide, the working linear ranges were set conservatively high to match the naturally high abundance of these analytes in the *P. perfoliatum* matrix. The limit of detection (LOD) and limit of quantification (LOQ) ranges were 0.6–6.3 μg/L and 2.1–19.5 μg/L, respectively. The relative standard deviation (RSD) values for precision were 1.4–4.2%. The recoveries of the 11 compounds within reasonable ranges were 94.4–102.8%. The results indicate that the established method is suitable for the quantitative analysis of the 11 compounds in *P. perfoliatum*. The representative chromatograms of samples and mixed standards are shown in Figure 3a,b.

#### 2.3.2. Quantitative Determination of 11 Major Components

**Figure 4:**
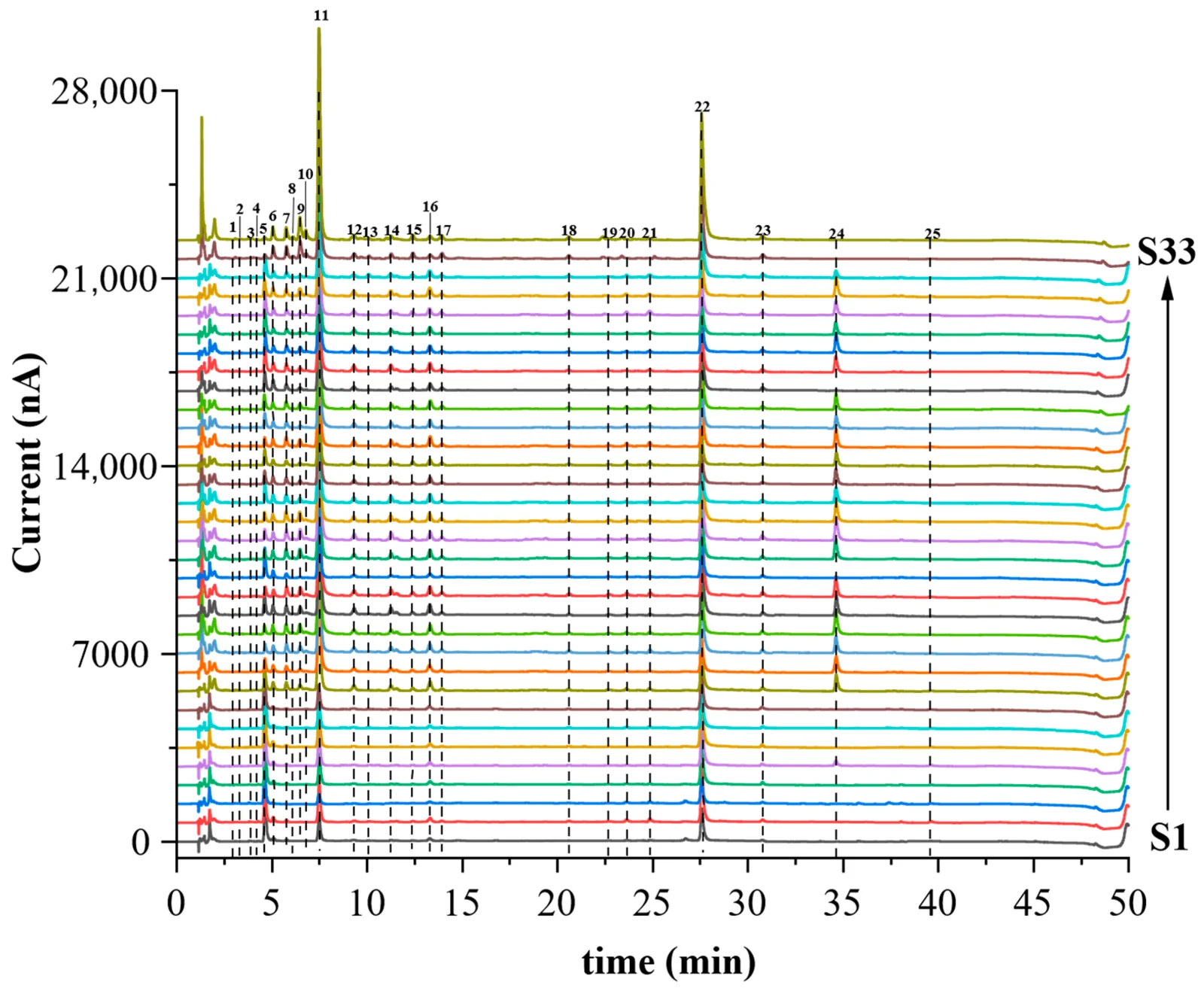
Overlaid HPLC-ECD chromatograms showing high similarity in peak profiles of 25 common characteristic peaks.

In 33 batches of *P. perfoliatum* from different sources, 25 peaks identified by UPLC-Orbitrap-MS/MS appeared in each sample, representing common peaks, as shown in Figure 4. The 25 peaks, labelled CP1 to CP25, are respectively: gallic acid, dihydroxybenzoic-acid-glucoside, procyanidin B isomer 1, *cis*-caffeoyltartaric acid, Trp, *trans*-caffeoyltartaric acid, catechin, *p*-coumaroyltartaric acid isomer 1, procyanidin B isomer 2, fertaric acid isomer 1, tartroylcaffeic acid, procyanidin C isomer 1, chlorogenic acid, *p*-coumaroyltartaric acid isomer 2, epicatechin, procyanidin B2, dihydroxyflavanone-glucoside, galloylcatechin, kaempferol-3-*O*-arabinoside, taxifolin, chicoric acid, quercetin-3-*O*-*β*-*D*-glucuronide, 1,5-dicaffeoylquinic acid, dihydro-tri-*O*-*p*-coumaroylsucrose, quercetin.

**Figure 5:**
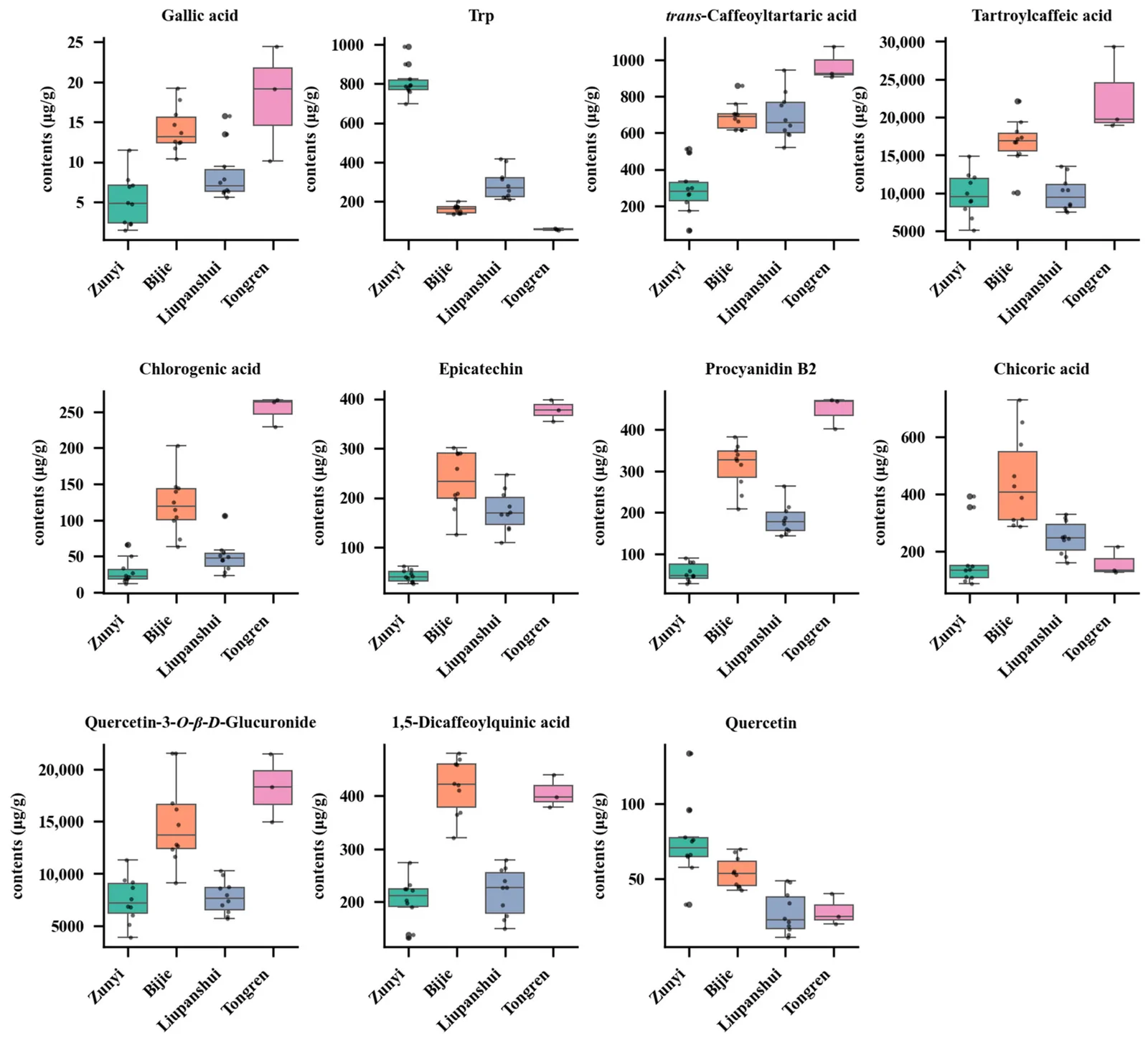
Comparative analysis of 33 batches of *P. perfoliatum* from four geographical origins. Box plot illustrating the distribution, median and variation range of contents (μg/g) of the 11 quantified core antioxidant compounds.

These 25 peaks accounted for a high proportion of the total peak area, while the remaining peaks had relatively small areas. This indicates that the distribution of *P. perfoliatum* is relatively concentrated across different batches, demonstrating good consistency. Meanwhile, the 11 compounds determined were those with larger peak areas in *P. perfoliatum.* The content of these 11 compounds in different batches of samples is presented in Table S1 (Supplementary Material) and Figure 5. The results show that all 11 compounds are present across all batches, although their concentrations vary. It should be noted that due to differences in batches and harvesting locations, the content of various components exhibits significant variations.

Furthermore, factors affecting the accumulation of active components, such as altitude, UV intensity, diurnal temperature variation, and soil conditions, vary. Consequently, there are notable differences in content levels among samples from different sources. In terms of average content, tartroylcaffeic acid was the most abundant component (5101.7–29,296.2 μg/g), followed by *trans*-caffeoyltartaric acid (66.0–1072.9 μg/g) and chicoric acid (97.8–727.4 μg/g). Among the flavonoids, quercetin-3-*O*-*β*-*D*-glucuronide had the highest concentration: 3876.9–21,535.2 μg/g. Of all compounds, gallic acid had the lowest concentration: 1.5–24.4 μg/g. The content ranges of other components were as follows: Trp (51.4–989.9 μg/g), chlorogenic acid (12.2–266.2 μg/g), epicatechin (26.7–397.7 μg/g), procyanidin B2 (28.3–471.3 μg/g), 1,5-dicaffeoylquinic acid (133.1–480.3 μg/g) and quercetin (10.5–133.9 μg/g).

Quercetin-3-*O*-*β*-*D*-glucuronide is a major flavonoid in *P. perfoliatum*, previously reported at 0.06–2.09% in 28 samples from different regions of China [45]. Our results (0.39–2.15%) are similar to this range. This compound was the most abundant flavonoid in our samples. Another key flavonoid, quercetin, was detected in our samples at 10.5–133.9 μg/g. A recent study covering 15 geographical origins reported quercetin contents of 15.67–50.19 μg/g [6], whereas the quercetin content in this study was 10.5–133.9 μg/g. The larger variation in our samples may reflect the diverse environmental conditions of the four collection regions. Notably, the previous study reported that quercetin was not detected in some samples, indicating that specific environmental conditions can completely inhibit its accumulation. Temperature may be a key factor affecting flavonoid accumulation; our samples were collected from high-altitude areas in August, when the temperature was relatively high, which may have led to a wider range of quercetin in this study. For gallic acid, our determined range (1.5–24.4 μg/g) is higher than the 0.49–2.21 μg/g reported by Zhang et al. [6] for samples from 15 sources. This difference may be attributed to differences in extraction solvents, sample preparation, and HPLC conditions.

Notably, the high abundance of *trans*-caffeoyltartaric acid (66.0–1072.9 μg/g) and tartroylcaffeic acid (5101.7–29,296.2 μg/g) identified in our study suggests that these compounds may be important constituents of *P. perfoliatum*, especially in Tongren samples (S31–S33). Chicoric acid is a caffeoyltartaric acid derivative known for its antioxidant and antiviral activities, and it has not been previously reported in metabolite studies of *P. perfoliatum*; its detected content in this study was 97.8–727.4 μg/g. These results significantly expand the known chemical profile of *P. perfoliatum* and provide a basis for future studies on its pharmacological activities.

### 2.4. Cluster Heatmap

**Figure 6:**
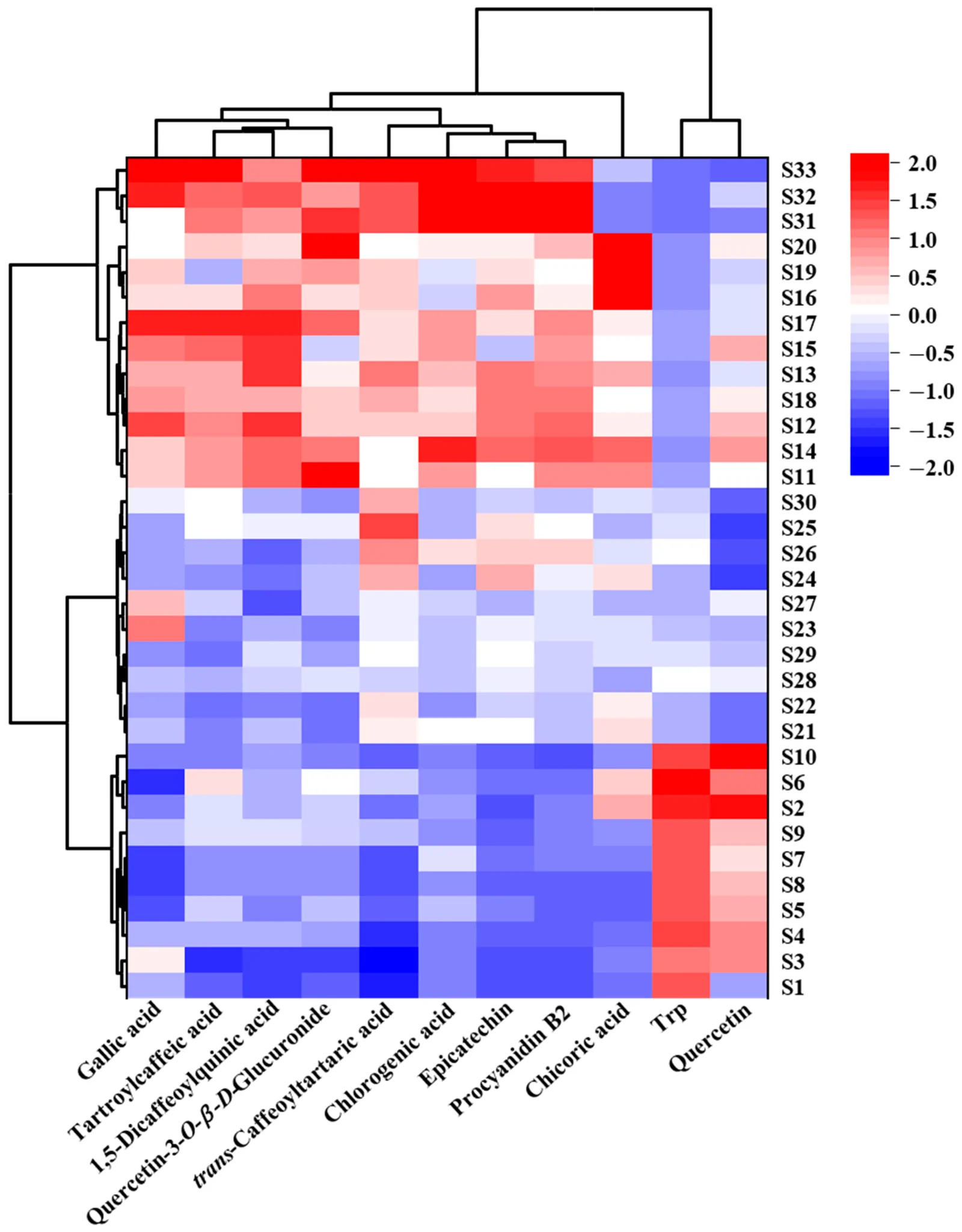
Cluster heatmap of 33 *P. perfoliatum* samples based on the contents of 11 quantified compounds.

Cluster heatmap was constructed based on quantitative results of the 11 target compounds to visualize the chemical characteristic differences among 33 batches of *P. perfoliatum* from different geographical origins. As shown in Figure 6, all samples were classified into four clusters: S1–S10 formed one cluster, S11–S20 formed another cluster, S21–S30 formed the third cluster, and S31–S33 formed the fourth cluster. The cluster assignment of samples was determined by their respective metabolic profiles. Among them, samples S1–S10 exhibited specific enrichment of Trp and quercetin alongside low accumulation of most phenolic acids and procyanidins, which distinguished them from the other samples. Samples S11–S20 displayed a metabolic signature with elevated contents of gallic acid, tartroylcaffeic acid, chlorogenic acid, procyanidin B2 and chicoric acid, forming an independent cluster. For samples S21–S30, the overall abundance of all quantified metabolites remained at a low level, making them a separate cluster. Samples S31–S33 were differentiated from other batches due to the high accumulation of multiple phenolic substances including gallic acid, *trans*-caffeoyltartaric acid and chicoric acid. Taken together, the divergence in metabolic profiles among samples fundamentally accounted for the four-cluster separation observed in this study.

Samples from the same geographical origin were clustered into independent groups, indicating the consistent internal quality of *P. perfoliatum* from identical habitats. Samples from the same source clustering into a single group may be attributed to similar natural conditions such as soil, climate, and elevation, resulting in clustering patterns in their chemical composition profiles. This reflects the synergistic regulatory role of geographic environmental factors on regional plant growth processes. The underlying causes of regional differences may stem from variations in the accumulation patterns of secondary metabolites resulting from the synergistic interaction of multiple factors [46]. On the one hand, abiotic factors such as soil trace element composition, hours of sunlight, and annual accumulated temperature directly influence the biosynthetic pathways of characteristic compounds like flavonoids and terpenoids by regulating the activity of key enzymes [47,48]. On the other hand, region-specific microbial community structures and their interactions with roots may induce plants to produce differential defensive metabolites. This explains why some samples from geographically distant locations cluster together due to similar biotic stress environments [49]. This coupling effect of ecological factors and metabolic networks indicates that the spatial differentiation of chemical components in *P. perfoliatum* is not only a representation of plant adaptation to the environment, but also a concrete manifestation of how geographic habitats drive quality formation through metabolic regulation [50,51].

### 2.5. Principal Component Analysis

To explore the overall chemical variation among *P. perfoliatum* samples from four geographical origins and to preliminarily visualize the sample clustering pattern, the contents of 11 quantified compounds in 33 batches of *P. perfoliatum* were selected as variables for principal component analysis (PCA). Three principal components were extracted according to the eigenvalue criterion (>1). PC1 had an eigenvalue of 6.849 and a variance explanation rate of 62.3%; PC2 had an eigenvalue of 1.482 and a variance explanation rate of 13.5%; and PC3 had an eigenvalue of 1.001 and a variance explanation rate of 9.1%. The cumulative variance of the first three principal components was 84.9%, meaning that these three principal components together explained 84.91% of the information in the original 11 variables and lost only 15.1% of the information.

**Figure 7:**
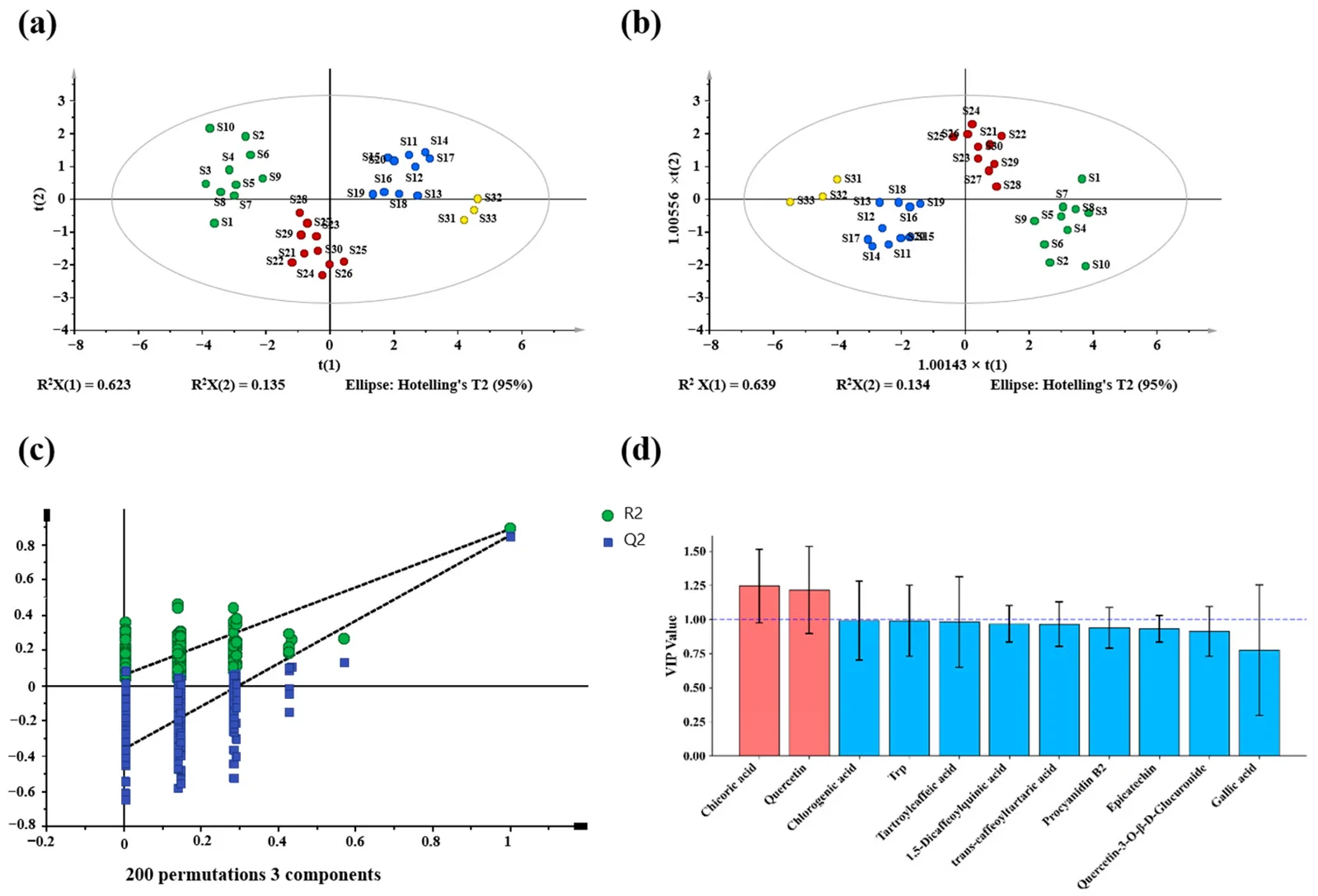
Multivariate statistical cluster analysis of 33 batches of *P. perfoliatum* samples. (**a**) PCA score plot, *R*^2^*X*(1) = 0.623 and *R*^2^*X*(2) = 0.135 represent the contribution rates of the first and second principal components; (**b**) OPLS-DA score plot, *R*^2^*X*(1) = 0.639 and *R*^2^*X*(2) = 0.134 represent the individual explained variances of the first and second predictive components, respectively; (**c**) 200-iteration permutation test of the OPLS-DA model; (**d**) variable importance in projection (VIP) bar plot of the OPLS-DA model.

By calculating the loading values of each variable, the key metabolites driving sample variation were screened. As shown in Table 3, PC1 was mainly contributed by positive-loading variables procyanidin B2, epicatechin, chlorogenic acid, 1,5-dicaffeoylquinic acid, and *trans*-caffeoyltartaric acid and negative-loading variable Trp; PC2 was dominated by variable quercetin; and PC3 was mainly driven by variable chicoric acid. In terms of group separation, PC1 clearly separated Zunyi (S1–S10) and Bijie (S11–S20) from Liupanshui (S21–S30) and Tongren (S31–S33): the former were distributed on the negative side of PC1, whereas the latter were on the positive side, indicating that PC1 mainly reflected differences in the contents of key compounds among these regions. Specifically, the positive-loading variables on PC1 (procyanidin B2, epicatechin, chlorogenic acid, 1,5-dicaffeoylquinic acid, and *trans*-caffeoyltartaric acid) were present at higher levels in samples from Liupanshui and Tongren, whereas Trp showed the opposite trend. PC2 was mainly dominated by variable quercetin, further distinguishing Tongren samples from those of other regions; PC3 was driven by variable chicoric acid, explaining subtle chemical differences among some samples. As shown in the PCA score plot (Figure 7a), the 33 batches of samples were divided into four groups, and all 33 samples fell within the 95% confidence ellipse with no outliers, which was highly consistent with the cluster heatmap results in Figure 6, further confirming the significant differences in chemical composition among samples from different origins. These results indicate that PCA not only achieved dimensionality reduction but also revealed the key compounds responsible for the grouping of samples from different geographical origins.

### 2.6. Orthogonal Partial Least Squares-Discriminant Analysis

To investigate the influence of geographical origin on the chemical profile of *P. perfoliatum* and to screen the key differential compounds responsible for geographical discrimination, the contents of 11 quantified compounds were selected as variables for orthogonal partial least squares-discriminant analysis (OPLS-DA), with sampling locations set as grouping indicators. The model parameters *R*^2^*X*(cum), *R*^2^*Y*(cum), and *Q*^2^(cum) describe the cumulative goodness of fit and predictive ability of the OPLS-DA model, respectively. An *R*^2^*X*(cum) value closer to 1 indicates a more stable model, while a higher *R*^2^*Y*(cum) reflects stronger explanatory power. All samples fell within the 95% confidence ellipse, and a *Q*^2^(cum) value greater than 0.5 indicates good predictive ability. As shown in Figure 7b, *R*^2^*X*(1) = 0.639 and *R*^2^*X*(2) = 0.134 represent the individual explanation rates of the first and second predictive components displayed on the horizontal and vertical axes of the score plot, respectively. The cumulative explanation rates were *R*^2^*X*(cum) = 0.864 and *R*^2^*Y*(cum) = 0.769, and the cross-validated *Q*^2^(cum) was 0.686. The OPLS-DA model exhibited satisfactory explanatory and predictive performance. All three statistical parameters were higher than the commonly accepted threshold of 0.5 and showed no obvious deviation, demonstrating that the constructed model avoided underfitting and overfitting and could reliably characterize the chemical differences in *P*. *perfoliatum* samples from the four geographical origins. The OPLS-DA score plot presented tight intra-group aggregation and distinct inter-group separation among samples from the four regions. Each geographical group occupied an independent and compact distribution area in the two-dimensional scoring space. No outlier samples were detected, which further verified the batch consistency of experimental samples and the good reproducibility of analytical data.

To eliminate overfitting induced by redundant variables and spurious correlations, a 200-iteration permutation test was performed for model validation. As illustrated in Figure 7c, the intercept values of *R*^2^ and *Q*^2^ were 0.061 and −0.359, respectively, verifying that the established OPLS-DA model was free of overfitting. On the basis of the validated reliable model, variable importance in projection (VIP) values were calculated to screen the core differential compounds contributing to geographical classification. As shown in Figure 7d, using VIP > 1.0 as the threshold, two differential markers were screened out, namely chicoric acid (VIP = 1.245) and quercetin (VIP = 1.215). Several other compounds had VIP values close to 1.0, including chlorogenic acid (VIP = 0.992), Trp (VIP = 0.991), tartroylcaffeic acid (VIP = 0.981), 1,5-dicaffeoylquinic acid (VIP = 0.967), *trans*-caffeoyltartaric acid (VIP = 0.965), procyanidin B2 (VIP = 0.938), epicatechin (VIP = 0.932), quercetin-3-*O*-*β*-*D*-glucuronide (VIP = 0.912), gallic acid (VIP = 0.775); all of these values were below 1.0. These key differential metabolites serve as effective chemical fingerprints for geographical traceability of *P. perfoliatum*. Furthermore, owing to their well-reported antioxidant activities, they are promising candidate markers for quality assessment and targeted monitoring of *P. perfoliatum* in future investigations.

## 3. Materials and Methods

### 3.1. Materials

#### 3.1.1. Plant Source

Fresh aerial parts of *P. perfoliatum* were collected in August. A total of 33 independent samples were obtained from different towns across four different regions of Guizhou Province. The plant material was identified by Dr. Lingjie Meng (Zunyi Medical University, Zunyi, Guizhou Province, China). Each sample has a voucher specimen record and is deposited in Room 2D-5, Research Building, Zunyi Medical University. Detailed information is provided in Table 4.

#### 3.1.2. Reagents and Standards

Gallic acid, chlorogenic acid, Trp, epicatechin, and quercetin were purchased from Aladdin Biochemical Technology Co., Ltd. (Shanghai, China); procyanidin B2, quercetin-3-*O*-*β*-*D*-glucuronide, 1,5-dicaffeoylquinic acid, and *trans*-caffeoyltartaric acid were purchased from Pufei Biotechnology Co., Ltd. (Chengdu, China). Chicoric acid was supplied by Tokyo Chemical Industry (TCI). All reference standards above had a purity of ≥98%. The tartroylcaffeic acid was self-prepared from the aerial parts of *P. perfoliatum* and verified by LC-HRMS (Figure S2). The purity was confirmed to exceed 98% via peak area normalization.

Methanol (HPLC grade), acetonitrile (HPLC grade), ABTS (analytical grade), and DPPH (analytical grade) were purchased from Aladdin Biochemical Technology Co., Ltd. Ultra-pure water was used throughout the experiment.

#### 3.1.3. Preparation of Sample and Standard Solutions

The aerial parts of *P. perfoliatum* were harvested in August, which is the optimal harvesting period. The samples were dried at 45 °C, pulverized, and passed through a 60-mesh sieve, and then stored at 4 °C until use. For sample preparation, 0.48 g of accurately weighed sample powder was extracted with 80% methanol. The solid–liquid ratios were set at 1 g:5 mL for LC×LC analysis and 1 g:10 mL for HPLC-ECD quantitative analysis. Ultrasonic extraction was performed at 30 °C and 40 kHz for 30 min. The extract was centrifuged at 9000 rpm for 5 min and filtered through a 0.22 μm microporous membrane to obtain the test solution. The extract was directly used for subsequent analysis.

An appropriate amount of reference standards, including gallic acid, Trp, chlorogenic acid, epicatechin, quercetin, quercetin-3-*O*-*β*-*D*-glucuronide, tartroylcaffeic acid, *trans*-caffeoyltartaric acid, chicoric acid, procyanidin B2, and 1,5-dicaffeoylquinic acid in appropriate amounts and dissolved in methanol to prepare standard stock solutions with a concentration of 1.0 mg/mL. The prepared stock solutions were stored at −20 °C for subsequent use. Before use, they were diluted with 80% methanol to prepare mixed standard solutions at required concentrations.

### 3.2. Methods

#### 3.2.1. LC×LC Separation and Component Identification

The analytical workflow was designed as follows: ^1^D-LC fractionation was performed using a semi-preparative phenyl-hexyl column, with fractions collected every 0.5 min. Each fraction was then subjected to two parallel analyses: (1) ^2^D-LC with ECD for antioxidant component screening and chromatographic peak capacity enhancement, and (2) UHPLC-Orbitrap-MS/MS for structural identification.

#### 3.2.2. First-Dimension LC Conditions

An e2695 HPLC system (Waters Corporation, Milford, MA, USA) was used with a Phenyl-Hexyl C18 column (10 mm × 150 mm, 5 μm, Welch Materials, Shanghai, China) as the stationary phase for the ^1^D-LC. The mobile phase consisted of 0.1% formic acid aqueous solution (A) and methanol (B). The gradient elution program was set as follows: 0–7 min, 5%→15% B; 7–35 min, 16%→28% B; 35–40 min, 28%→40% B; 40–52 min, 40%→65% B; 52–56 min, 65%→95% B. The flow rate was 2.5 mL/min, the injection volume was 80 μL, the column temperature was 35 °C, and the detection wavelength was 280 nm. Fractions were collected every 0.5 min for ^2^D-LC separation, LC-MS/MS identification, and free radical scavenging capacity assays.

#### 3.2.3. Second-Dimension LC Conditions

Second-dimension separation was performed on an UltiMate 3000 bio-RS UHPLC system (Thermo Fisher Scientific, Waltham, MA, USA) equipped with an ECD-3000 RS electrochemical detector (coulometric detection cell). The stationary phase was an ACQUITY UPLC BEH C18 column (2.1 mm × 100 mm, 1.7 µm, Waters Corporation, Milford, MA, USA), and the mobile phase consisted of acetonitrile (A) and a 50 mmol/L citric acid-potassium citrate solution at pH 2.65 (B). Different gradients were used for fractions from the first-dimension: F1 (5–14 min): 0–6 min, 2.5%→15% A; F2 (14.5–30 min): 0–6 min, 10%→25% A; F3 (30.5–43 min): 0–6 min, 15%→40% A; F4 (43.5–60 min): 0–6 min, 20%→45% A. After each gradient elution procedure, the mobile phase was returned to the initial ratio and balanced for 2.5 min to ensure column stability for the next injection. The flow rate was 0.35 mL/min, the injection volume was 10 μL, the column temperature was 45 °C, the sample tray temperature was 12 °C, and the detection potential was 600 mV.

#### 3.2.4. UHPLC-Orbitrap-MS/MS Conditions

UHPLC-Orbitrap-MS/MS analysis was performed using a Vanquish F UPLC system coupled with an Orbitrap Exploris 120 mass spectrometer (Thermo Fisher Scientific, Waltham, MA, USA).

Chromatographic separation was performed on an ACQUITY UPLC BEH C18 column (2.1 mm × 100 mm, 1.7 µm, Waters), which was identical to that used for ^2^D-LC-ECD. To ensure accurate peak matching between electrochemical detection and mass spectrometry for each fraction, the gradient elution profile (time-dependent mobile phase ratio curve) for F1–F4 fractions were fully consistent with that used in the second-dimension LC-ECD. Only the mobile phase was adjusted to adapt to electrospray ionization requirements: acetonitrile (A) and 0.1% formic acid aqueous solution (B) were used to replace the non-volatile citrate buffer system, so as to avoid ion suppression and ion source contamination. The flow rate, column temperature and injection volume were consistent with those of ^2^D-LC-ECD. The replacement of citrate with 0.1% formic acid for MS detection induced changes in pH and ionic strength to a certain extent, leading to slight retention time shifts of some chromatographic peaks. Therefore, peak matching was preliminarily confirmed based on retention time (both absolute retention time and relative retention time referenced to adjacent large-volume chromatographic peaks) and further validated by accurate mass, MS/MS fragments, and ECD responses of fractions. Since the chemical constituents in fractions were substantially reduced compared with the original sample, matrix interference was relatively low, enabling relatively accurate matching of these chromatographic peaks. Unresolved isomers were annotated as tentative identifications or isomer groups in Table 2.

The ion transfer tube temperature was 320 °C, and the capillary temperature was 350 °C. The sheath gas pressure, auxiliary gas pressure and sweep gas pressure were 35 Arb, 10 Arb and 3 Arb, respectively. Mass spectrometry detection was operated in negative mode with a spray voltage of 2500 V and an RF voltage of 70 V. Data-dependent acquisition (DDA) mode was applied for compound identification. The full-scan MS scanning range was *m*/*z* 100–1500 with a resolution of 60,000. The MS^2^ collision energy was set at 20%, 40% and 60% with a resolution of 15,000. Precursor ions and fragment ions of compounds in collected fractions were acquired. Data acquisition and processing were performed using TraceFinder 5.2.0 software. Compounds were identified by matching against databases in Compound Discoverer 3.4.0 and Sirius 6.3.3 software.

#### 3.2.5. Quantitative Analysis of the Target Components

Chromatographic Parameters of LC-ECD for Quantitative Assay

LC-ECD was used to determine the contents of major components in 33 batches of *P. perfoliatum* samples from different habitats. The instrument and mobile phase were the same as those used for ^2^D-LC. An XBridge BEH Shield RP18 column (3.0 mm × 150 mm, 2.5 μm, Waters Corporation, Milford, MA, USA) was used as the stationary phase. Gradient elution was as follows: 0–17 min, 5%→12% A; 17–40 min, 12%→30% A; 40–45 min, 30%→60% A; 45–47 min, 60%→95% A. The ECD potential was 600 mV, flow rate was 0.6 mL/min, column temperature was 45 °C, sample tray temperature was 12 °C, and injection volume was 1 μL.

Method Validation

Linearity, LOD, LOQ, precision, and recovery were investigated. Mixed standard solutions with a series of concentrations were analyzed under the optimized chromatographic conditions. Calibration curves were established with concentration (*X*) as the abscissa and characteristic peak area (*Y*) as the ordinate to calculate regression equations. LOD and LOQ were calculated at signal-to-noise ratios (S/N) of 3 and 10, respectively. The precision was evaluated by six consecutive injections of mixed standard solutions. Accuracy was evaluated using the spiked recovery method. Known concentrations of reference standards were added into *P. perfoliatum* extract, and six parallel spiked samples were analyzed to calculate the average recovery and RSD.

#### 3.2.6. In Vitro Antioxidant Activity Assay of ^1^D-LC Fractions

ABTS Radical Scavenging Assay

The assay was performed with minor modifications according to Do et al. [52]. Briefly, 100 μL of each ^1^D-LC fraction collected at different time points was dispensed into a 96-well plate, followed by 200 μL of ABTS (0.1 mmol/L). After mixing and incubating in the dark for 30 min, the absorbance was measured at 734 nm using a microplate reader. The ABTS scavenging capacity was calculated using the calibration curve (y = −0.0054x + 0.3272, *R*^2^ = 0.9941) of Trolox, and the results were expressed as µg Trolox equivalents (TE)/mL.

DPPH Radical Scavenging Assay

Following the method of Duguma et al. [53] with slight modifications, 100 μL of ^1^D-LC fractions collected at different time points were dispensed into a 96-well plate. Then, 200 μL of DPPH (0.1 mmol/L) was added, mixed well, and allowed to react in the dark for 15 min. Absorbance was measured in triplicate at 517 nm using a microplate reader. The DPPH scavenging capacity was calculated using the calibration curve (y = −0.0025x + 0.3256, *R*^2^ = 0.9987) of Trolox, and the results were expressed as µg TE/mL.

#### 3.2.7. Multivariate Statistical Analysis

The contents of 11 quantified compounds from 33 batches of samples were used as variables. A cluster heatmap was constructed using Origin 2021, with Ward’s method as the merging rule. PCA and OPLS-DA were performed using Origin 2021 and SIMCA 14.1.0, respectively. A 200-iteration permutation test was applied to validate the OPLS-DA model, and VIP values were calculated.

## 4. Conclusions

In conclusion, an integrated LC×LC-ECD/UHPLC-Orbitrap-MS/MS strategy was established for activity-guided antioxidant screening in *P. perfoliatum*. This approach effectively overcomes the separation limitations of 1D-LC and enables the correlation of electrochemical responses with the separated fractions, thereby facilitating the rapid screening of potential phenolic antioxidants. The novelty of this work lies in the integrated analytical strategy and its application to activity-guided screening. Using this strategy, the relative contributions of the fractions containing the identified phenolic compounds to the in vitro chemical radical scavenging capacity were systematically evaluated, and the 11 major compounds were quantified. These findings provide a reliable methodological basis for the quality evaluation and resource development of *P. perfoliatum*, and also offer a useful reference for the systematic screening of antioxidants in other complex medicinal and edible plants.

## Figures and Tables

**Table 1:** Chemical constituents of *P. perfoliatum* identified by UPLC-Orbitrap MS/MS.

Number	Precursorions (*m*/*z*)	Fragment Ions (*m*/*z*)	Compounds	Molecular Formula	Confidence Level
1	331.0664	169.0140, 151.0031, 125.0243	Glucogallin isomer 1	C_13_H_16_O_10_	Level II
2	331.0669	169.0145, 151.0032, 125.0247	Glucogallin isomer 2	C_13_H_16_O_10_	Level II
3	331.0667	169.0141, 151.0036, 125.0244	Glucogallin isomer 3	C_13_H_16_O_10_	Level II
4	331.0668	169.0146, 151.0040, 125.0249	Glucogallin isomer 4	C_13_H_16_O_10_	Level II
5	331.0667	169.0143, 151.0042, 125.0244	Glucogallin isomer 5	C_13_H_16_O_10_	Level II
6	169.0140	125.0243	Gallic acid	C_7_H_6_O_5_	Level I
7	315.0718	153.0558, 123.0451, 108.0216	Dihydroxybenzoic acid *O*-glucoside isomer 1	C_13_H_16_O_9_	Level II
8	331.1032	153.0192, 168.0425	Koaburaside	C_14_H_20_O_9_	Level II
9	329.0876	116.0505, 74.0247, 142.0661, 159.0927	Vanillic acid *O*-glucopyranoside	C_14_H_18_O_9_	Level II
10	577.1346	407.0766, 289.0742, 125.0250	Procyanidin B isomer 1	C_30_H_26_O_12_	Level II
11	315.0718	153.0558, 123.0451, 108.0216	Dihydroxybenzoic acid *O*-glucoside isomer 2	C_13_H_16_O_9_	Level II
12	313.0927	107.0502, 151.0400	Hydroxyphenylacetic acid glucoside	C_14_H_18_O_8_	Level II
13	577.1345	407.0779, 289.0712, 125.0243	Procyanidin B isomer 2	C_30_H_26_O_12_	Level II
14	311.0405	179.0348, 149.0090, 135.0451	*cis*-Caffeoyltartaric acid	C_13_H_12_O_9_	Level II
15	203.0822	116.0505, 74.0247, 142.0661, 159.0927	Tryptophan (Trp)	C_11_H_12_N_2_O_2_	Level I
16	341.0872	161.0242, 179.0357, 133.0295	Caffeoyl-beta-*D*-glucose	C_15_H_18_O_9_	Level II
17	865.1984	575.1139, 161.0246, 287.0587	Procyanidin C isomer 1	C_45_H_38_O_18_	Level II
18	465.1033	341.0701, 303.0526, 275.4923, 153.0202	Isoglucodistylin	C_21_H_22_O_12_	Level II
19	289.0716	245.0838, 109.0294, 123.0456, 203.0732	Catechin	C_15_H_14_O_6_	Level II
20	311.0406	149.0091, 179.0349, 135.0452	*trans*-Caffeoyltartaric acid	C_13_H_12_O_9_	Level I
21	295.0457	163.0400, 149.0091, 119.0502	*p*-Coumaroyltartaric acid isomer 1	C_13_H_12_O_8_	Level II
22	295.0456	163.0399, 119.0502	*p*-Coumaroyltartaric acid isomer 2	C_13_H_12_O_8_	Level II
23	577.1344	407.0764, 289.0716, 125.0243	Procyanidin B isomer 2 (Procyanidin B2)	C_30_H_26_O_12_	Level I
24	311.0404	179.0347, 149.0090, 135.0450	Tartroylcaffeic acid	C_13_H_12_O_9_	Level II
25	353.0873	191.0559	Chlorogenic acid	C_16_H_18_O_9_	Level I
26	325.0926	119.0502, 163.0400	*p*-Coumaric acid glucoside isomer 1	C_15_H_18_O_8_	Level II
27	325.0562	193.0505, 179.0349, 149.0093, 135.0407	Fertaric acid isomer 1	C_14_H_14_O_9_	Level II
28	483.0772	439.0878, 125.0243, 125.0242	Di-*O*-galloyl-*β-D*-Glucopyranose	C_20_H_20_O_14_	Level II
29	729.1451	577.1361, 407.0776, 289.0714, 125.0242	2×(E)C-GA	C_37_H_30_O_16_	Level II
30	385.1134	223.0610, 179.0711, 165.0508, 149.0243	Sinapic acid glucoside isomer 1	C_17_H_22_O_10_	Level II
31	865.1980	577.1346, 407.0793, 289.0558, 161.0243	Procyanidin C isomer 2	C_45_H_38_O_18_	Level II
32	325.0924	119.0501, 163.0402	*p*-Coumaric acid glucoside isomer 2	C_13_H_12_O_8_	Level II
33	295.0455	149.0090, 163.0399, 119.0502	*p*-Coumaroyltartaric acid isomer 3	C_13_H_12_O_8_	Level II
34	355.1031	193.0505, 134.0373, 178.0270	Ferulic acid-*O*-*β*-*D*-Glucoside	C_16_H_20_O_9_	Level II
35	289.0715	245.0819, 109.0295, 123.0452	Epicatechin	C_15_H_14_O_6_	Level I
36	325.0926	119.0502, 163.0401	*p*-Coumaric acid glucoside isomer 3	C_13_H_12_O_8_	Level II
37	433.1136	271.0612, 229.0506, 177.0194	Dihydroxyflavanone-Glucoside	C_21_H_22_O_10_	Level II
38	325.0561	193.0512, 179.0345, 149.0097, 135.0403	Fertaric acid isomer 2	C_14_H_14_O_9_	Level II
39	385.1134	223.0610, 179.0711, 165.0508, 149.0243	Sinapic acid glucoside isomer 2	C_17_H_22_O_10_	Level II
40	325.0561	193.0513, 179.0351, 149.0095, 135.0403	Fertaric acid isomer 3	C_14_H_14_O_9_	Level II
41	325.0563	193.0509, 179.0352, 149.0090, 135.0410	Fertaric acid isomer 4	C_14_H_14_O_9_	Level II
42	319.0457	183.0298, 139.0400, 153.0193	Dihydroxybenzoyl-Trihydroxyphenylacetic isomer 1 1acid	C_15_H_12_O_8_	Level II
43	325.0563	193.0506, 179.0347, 149.0091, 135.0405	Fertaric acid isomer 5	C_14_H_14_O_9_	Level II
44	441.0820	289.0716, 169.0142, 125.0243	Galloylcatechin	C_22_H_18_O_10_	Level II
45	417.0825	285.0402, 147.0087	Kaempferol-3-*O*-arabinoside	C_20_H_18_O_10_	Level II
46	441.0823	289.0719, 169.0143, 125.0245	Teatannin	C_22_H_18_O_10_	Level II
47	319.0457	183.0298, 139.0400, 153.0193	Dihydroxybenzoyl-Trihydroxyphenylacetic isomer 2 1acid	C_15_H_12_O_8_	Level II
48	303.0508	285.0403, 125.0244	Taxifolin	C_15_H_12_O_7_	Level II
49	309.0614	147.0298, 129.0193	Caffeoylglutaric acid	C_14_H_14_O_8_	Level II
50	473.0723	311.0403, 179.0344	Chicoric acid	C_22_H_18_O_12_	Level I
51	455.0977	303.0872, 285.0768, 169.0142, 125.0243	Epicatechin 3-*O*-(4-*O*-Methylgallate)	C_23_H_20_O_10_	Level II
52	463.0874	316.0221, 271.0245	Myricitrin	C_21_H_20_O_12_	Level II
53	477.0663	301.0351, 300.0237, 151.0036	Quercetin-3-*O*-*β*-*D*-Glucuronide	C_21_H_18_O_13_	Level I
54	463.0873	300.0271, 301.0342	Hyperin	C_21_H_20_O_12_	Level II
55	477.0669	301.0347,300.0273, 151.0045	Quercetin-7-*O*-*β*-*D*-Glucuronide	C_21_H_18_O_13_	Level II
56	433.0767	300.0273, 301.0341, 271.0246, 255.0296	Avicularin	C_20_H_18_O_11_	Level II
57	461.0718	285.0401, 229.0503	Luteolin-7-*O*-Glucuronide	C_21_H_18_O_12_	Level II
58	447.0924	300.0273, 301.0341, 271.0246, 255.0298	Trifolin	C_21_H_20_O_11_	Level II
59	735.2128	559.1660, 175.0398, 160.0164	Helonioside B	C_34_H_40_O_18_	Level II
60	515.1196	353.0872, 191.0556, 179.0344	1,5-Dicaffeoylquinic acid	C_25_H_24_O_12_	Level I
61	819.2343	175.0399, 160.0164	Smiglaside C	C_38_H_44_O_20_	Level II
62	779.2182	633.1815, 615.1737, 145.0294, 117.0344	Dihydro-tri-*O*-*p*-coumaroylsucrose	C_39_H_40_O_17_	Level II
63	301.0355	179.0344, 151.0028	Quercetin	C_15_H_10_O_7_	Level I

**Table 2:** Linear regression, precision, and recovery of 11 quantitatively determined components in *P. perfoliatum*.

Compound	Linear Equation	*R* ^2^	Linear Range (μg/L)	LOQ (μg/L)	LOD (μg/L)	Precision (RSD%)	Average Recovery (%)	RSD of Recovery (%)
Gallic acid	y = 11.77x − 0.01	0.9995	100–5000	2.9	1.0	2.1	95.8	4.9
Trp	y = 26.48x + 1.02	0.9997	200–10,000	2.1	0.6	2.6	94.4	3.9
*trans*-Caffeoyltartaric acid	y = 5.12x + 0.11	0.9998	2000–100,000	8.6	2.5	3.0	101.4	5.7
Tartroylcaffeic acid	y = 5.57x + 0.41	0.9998	2000–100,000	13.5	3.2	1.4	98.9	4.9
Chlorogenic acid	y = 6.24x + 0.68	0.9996	200–20,000	6.4	1.8	1.6	97.6	2.6
Epicatechin	y = 8.94x + 0.99	0.9991	200–100,000	14.4	3.0	2.7	95.8	5.7
Procyanidin B2	y = 8.38x + 0.93	0.9997	200–10,000	6.9	2.8	3.9	94.1	4.2
Chicoric acid	y = 5.08x − 0.08	0.9994	5000–20,000	8.6	2.8	2.0	96.4	2.8
Quercetin-3-O-*β*-*D*-Glucuronide	y = 4.02x + 3.52	0.9993	2000–200,000	19.5	6.3	4.2	102.8	3.3
1,5-Dicaffeoylquinic acid	y = 6.67x + 0.39	0.9996	200–20,000	12.2	4.2	3.9	96.7	4.6
Quercetin	y = 6.48x − 0.73	0.9995	100–5000	4.6	1.4	1.9	95.4	3.1

Note: LOD and LOQ were calculated by the signal-to-noise ratio method according to the ICH *Q2*(*R*^2^) guideline (S/N = 3 for LOD and S/N = 10 for LOQ). The linear range represents the experimentally validated working concentration range, and all concentration points within this range meet the accuracy and precision requirements.

**Table 3:** PCA loadings of the 11 quantified compounds in *P. perfoliatum* on PC1, PC2, and PC3.

Component	PC1	PC2	PC3
Gallic acid	0.265	0.1201	0.021
Trp	−0.332	0.259	−0.190
*trans*-caffeoyltartaric acid	0.321	−0.322	0.021
Tartroylcaffeic acid	0.312	0.251	−0.243
Chlorogenic acid	0.3291	0.119	−0.342
Epicatechin	0.352	−0.180	−0.063
Procyanidin B2	0.374	−0.008	−0.119
Chicoric acid	0.157	0.172	0.868
Quercetin-3-*O*-*β*-*D*-Glucuronide	0.305	0.271	0.105
1,5-Dicaffeoylquinic acid	0.325	0.319	0.009
Quercetin	−0.152	0.707	−0.053

**Table 4:** Detailed sample information of 33 batches of *P. perfoliatum* from four producing areas in Guizhou Province.

Sample	Origin	Time	Coordinates	Elevation (m)	Soil	Temperature (°C)	Number
S1	Wuchuan, Zunyi	6 August 2025	107°43′ E, 28°42′ N	700	Yellow soil	24–28	WC202501
S2	Wuchuan, Zunyi	6 August 2025	107°33′ E, 28°51′ N	850	Yellow soil	24–28	WC202502
S3	Wuchuan, Zunyi	6 August 2025	107°51′ E, 28°18′ N	650	Yellow soil	24–28	WC202503
S4	Honghuagang, Zunyi	6 August 2025	106°56′ E, 27°35′ N	850	Yellow soil	24–30	HHG202501
S5	Honghuagang, Zunyi	6 August 2025	106°49′ E, 27°45′ N	900	Yellow soil	24–30	HHG202502
S6	Honghuagang, Zunyi	6 August 2025	106°47′ E, 27°49′ N	950	Yellow soil	24–30	HHG202503
S7	Honghuagang, Zunyi	6 August 2025	106°51′ E, 27°41′ N	880	Yellow soil	24–30	HHG202504
S8	Yuqing, Zunyi	6 August 2025	107°53′ E, 27°13′ N	680	Yellow soil	25–29	YQ202501
S9	Yuqing, Zunyi	6 August 2025	107°42′ E, 27°06′ N	620	Yellow soil	25–29	YQ202502
S10	Yuqing, Zunyi	6 August 2025	107°27′ E, 27°33′ N	800	Yellow soil	25–29	YQ202503
S11	Jinsha, Bijie	11 August 2025	106°27′ E, 27°18′ N	950	Yellow soil	22–26	JS202501
S12	Jinsha, Bijie	11 August 2025	106°21′ E, 27°15′ N	900	Yellow soil	22–26	JS202502
S13	Jinsha, Bijie	11 August 2025	106°00′ E, 27°42′ N	1100	Yellow soil	22–26	JS202503
S14	Hezhang, Bijie	11 August 2025	104°48′ E, 27°03′ N	1600	Yellow-brown soil	18–22	HZ202501
S15	Hezhang, Bijie	11 August 2025	104°36′ E, 26°54′ N	1800	Yellow-brown soil	18–22	HZ202502
S16	Hezhang, Bijie	11 August 2025	104°24′ E, 27°15′ N	1700	Yellow-brown soil	18–22	HZ202503
S17	Zhijin, Bijie	11 August 2025	105°39′ E, 26°36′ N	1300	Yellow soil	20–24	ZJ202501
S18	Zhijin, Bijie	11 August 2025	105°57′ E, 26°33′ N	1200	Yellow soil	20–24	ZJ202502
S19	Zhijin, Bijie	11 August 2025	105°45′ E, 26°51′ N	1450	Yellow soil	20–24	ZJ202503
S20	Zhijin, Bijie	11 August 2025	105°27′ E, 26°27′ N	1500	Yellow soil	20–24	ZJ202504
S21	Shuicheng, Liupanshui	15 August 2025	104°43′ E, 26°33′ N	1850	Yellow-brown soil	16–20	SC202501
S22	Shuicheng, Liupanshui	15 August 2025	104°33′ E, 26°25′ N	1600	Yellow-brown soil	16–20	SC202502
S23	Shuicheng, Liupanshui	15 August 2025	104°12′ E, 26°33′ N	1700	Yellow-brown soil	16–20	SC202503
S24	Panzhou, Liupanshui	15 August 2025	104°21′ E, 25°57′ N	1550	Yellow soil	18–22	PZ202501
S25	Panzhou, Liupanshui	15 August 2025	104°33′ E, 25°42′ N	1650	Yellow soil	18–22	PZ202502
S26	Panzhou, Liupanshui	15 August 2025	104°12′ E, 25°45′ N	1750	Yellow soil	18–22	PZ202503
S27	Liuzhi, Liupanshui	15 August 2025	105°25′ E, 26°18′ N	1350	Yellow soil	20–24	LZ202501
S28	Liuzhi, Liupanshui	15 August 2025	105°18′ E, 26°03′ N	1200	Yellow soil	20–24	LZ202502
S29	Liuzhi, Liupanshui	15 August 2025	105°36′ E, 26°09′ N	1250	Yellow soil	20–24	LZ202503
S30	Liuzhi, Liupanshui	15 August 2025	105°12′ E, 26°15′ N	1500	Yellow soil	20–24	LZ202504
S31	Songtao, Tongren	20 August 2025	108°23′ E, 28°15′ N	600	Red soil	26–30	ST202501
S32	Songtao, Tongren	20 August 2025	108°03′ E, 27°57′ N	500	Red soil	26–30	ST202502
S33	Songtao, Tongren	20 August 2025	108°57′ E, 28°15′ N	700	Red soil	26–30	ST202503

## Data Availability

The data used to support the findings of this study are available from the corresponding author upon request.

## Supplementary Materials

The following supporting information can be downloaded at: https://www.mdpi.com/article/10.3390/molecules31203612/s1, Figure S1: Stacked total ion chromatograms (TICs) of 63 annotated chromatographic peaks from high-resolution mass spectrometry; Figure S2: LC-HRMS profile of self-prepared tartroylcaffeic acid; Figure S3: LC-HRMS spectra of three isomers with molecular formula C_13_H_12_O_9_ detected in *P. perfoliatum*; Table S1: Contents of 11 major antioxidant components in 33 batches of *P. perfoliatum* (μg/g).
