# Supplementary material for: Screening of Phenolic Antioxidant Components in Polygonum perfoliatum L. Using LC×LC-ECD and UPLC-Orbitrap-MS/MS
Source: Molecules. 2026 Oct 10;31(20):3612. doi: 10.3390/molecules31203612 (PMC13707955; doi:10.3390/molecules31203612)
Supplement: Supplementary file 1 [file molecules-31-03612-s001.zip › Supplementary_FigureS3.pdf]

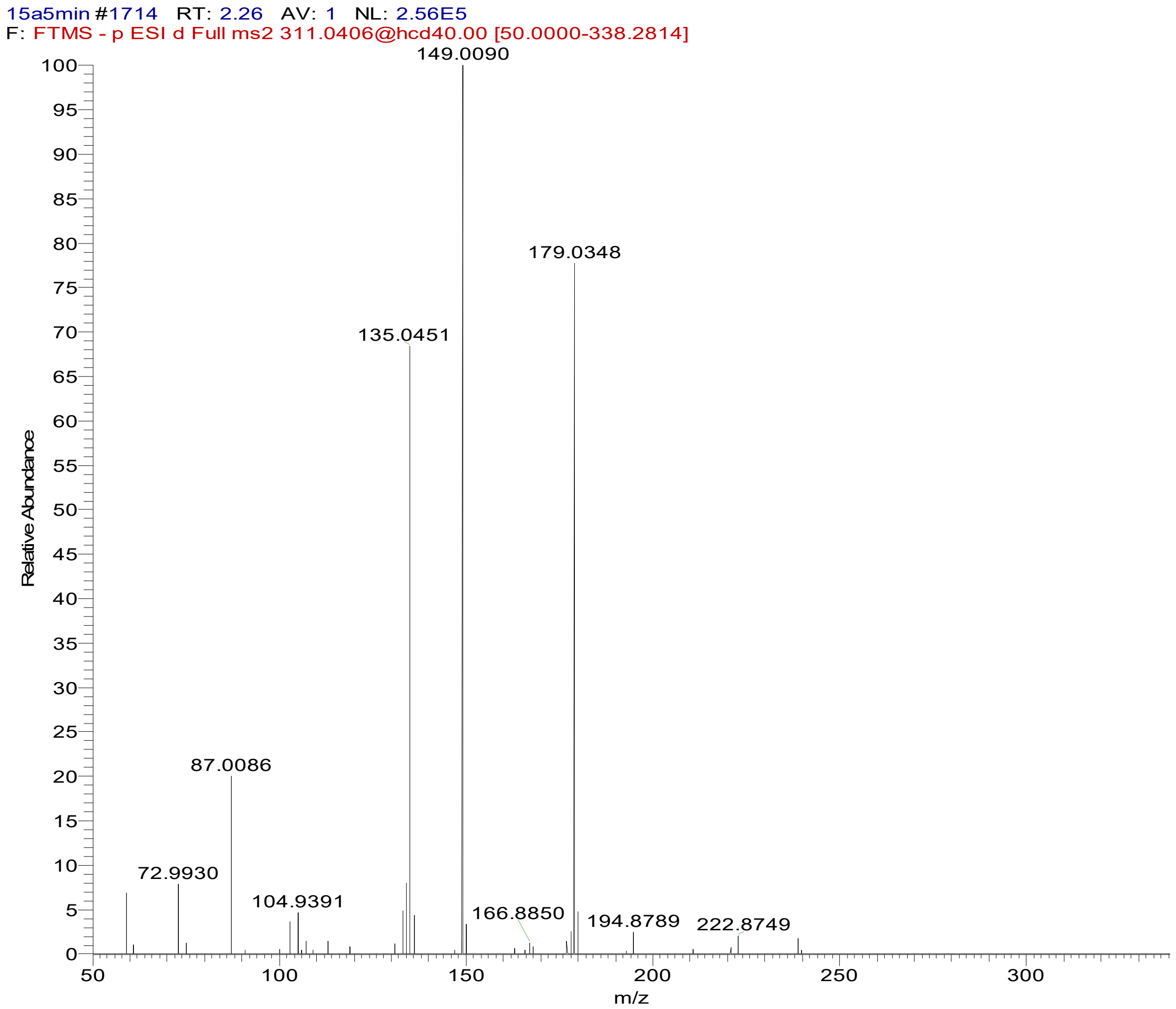

15a5min #1714 RT: 2.26 AV: 1 NL: 2.56E5
F: FTMS - p ESI d Full ms2 311.0406@hcd40.00 [50.0000-338.2814]
149.0090
179.0348
135.0451
87.0086
72.9930
104.9391
166.8850
194.8789
222.8749
Relative Abundance
m/z

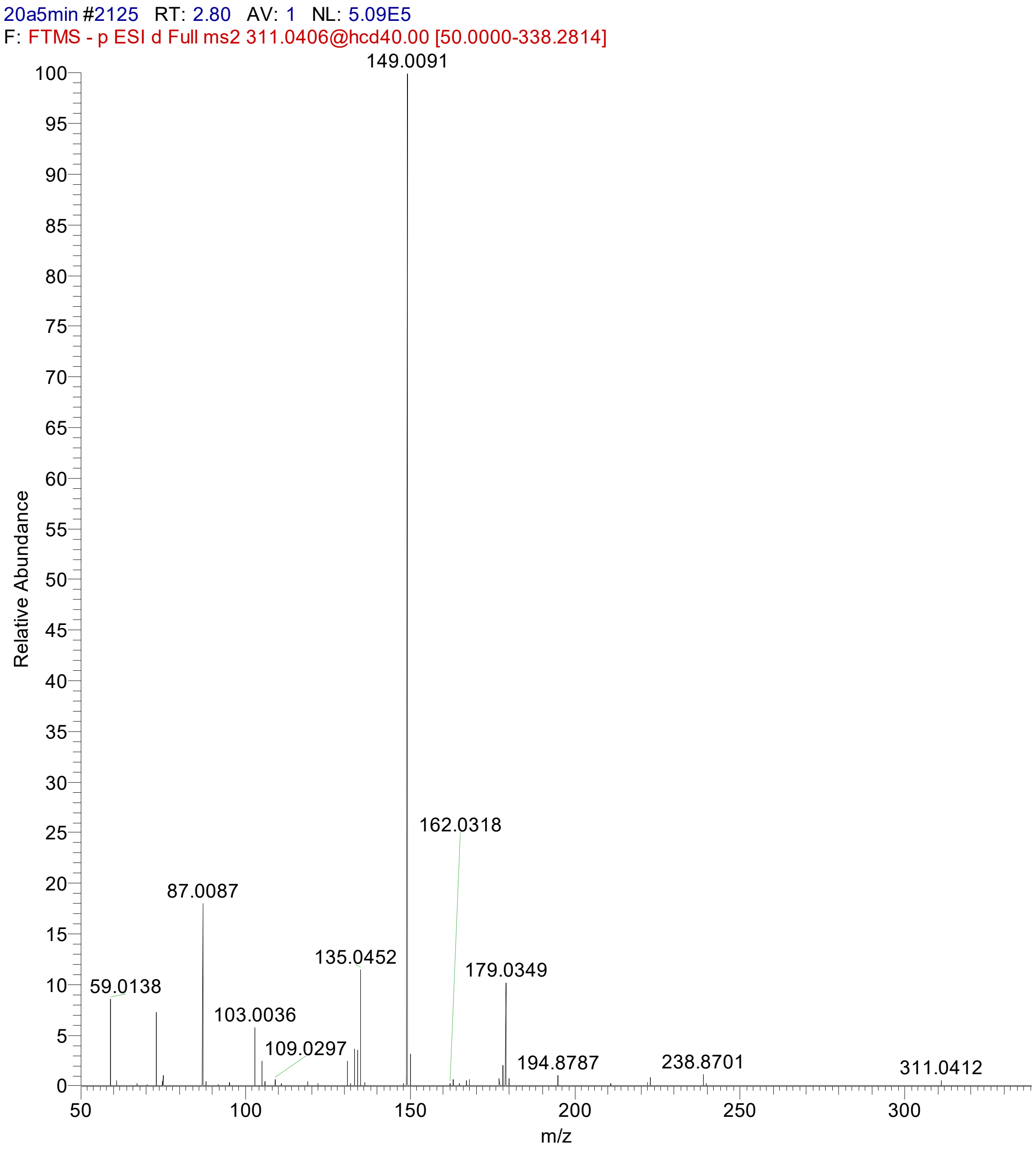

20a5min #2125 RT: 2.80 AV: 1 NL: 5.09E5
F: FTMS - p ESI d Full ms2 311.0406@hcd40.00 [50.0000-338.2814]
149.0091
162.0318
87.0087
135.0452
179.0349
59.0138
103.0036
109.0297
194.8787
238.8701
311.0412
Relative Abundance
m/z

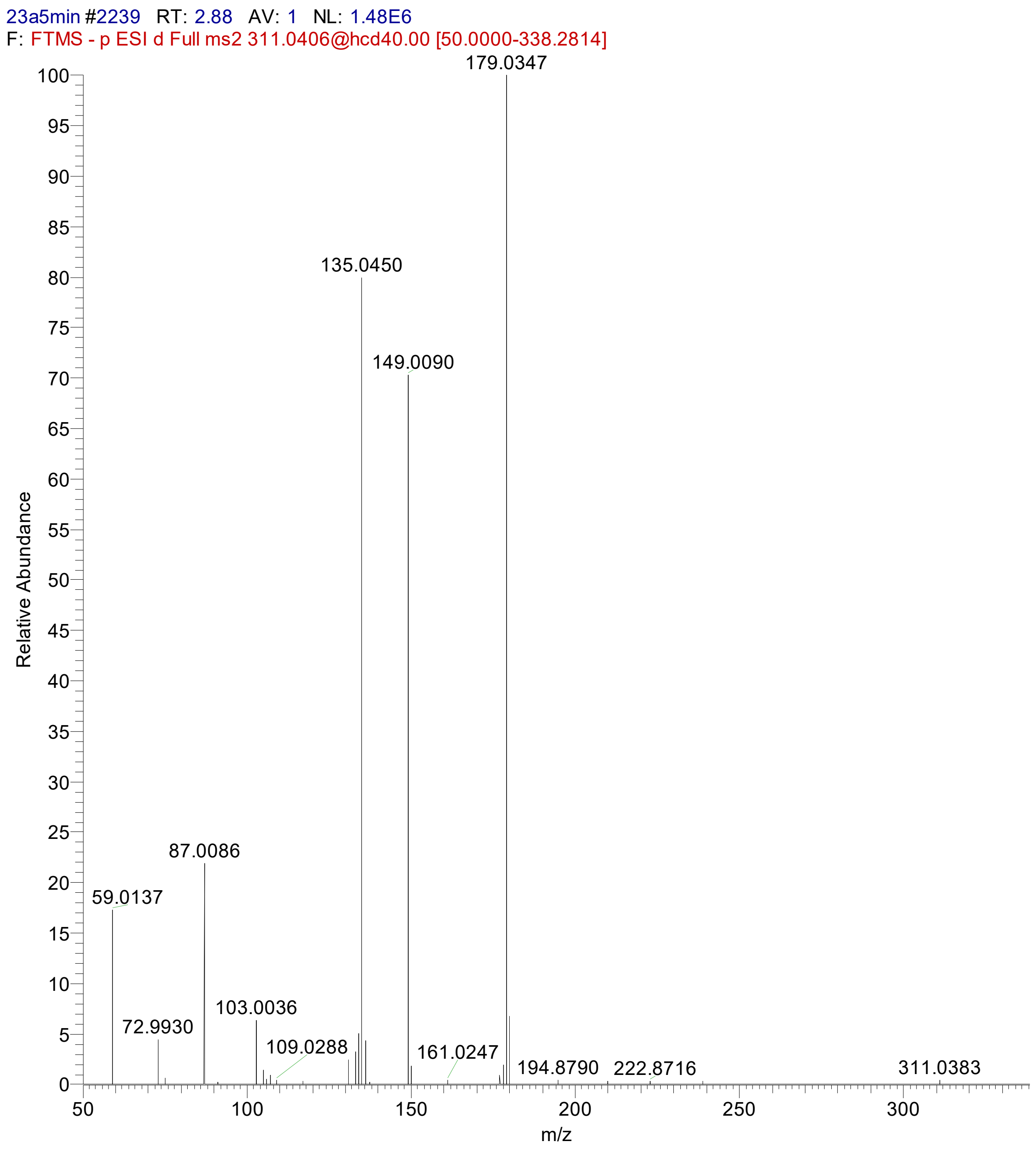

23a5min #2239 RT: 2.88 AV: 1 NL: 1.48E6
F: FTMS - p ESI d Full ms2 311.0406@hcd40.00 [50.0000-338.2814]
179.0347
135.0450
149.0090
87.0086
59.0137
103.0036
72.9930
109.0288
161.0247
194.8790
222.8716
311.0383
Relative Abundance
m/z
